# A Multivalent Vaccine Based on Ferritin Nanocage Elicits Potent Protective Immune Responses against SARS-CoV-2 Mutations

**DOI:** 10.3390/ijms23116123

**Published:** 2022-05-30

**Authors:** Seong A. Kim, Seohyun Kim, Gi Beom Kim, Jiyoung Goo, Nayeon Kim, Yeram Lee, Gi-Hoon Nam, Seungho Lim, Taeerk Kim, Ki Hwan Chang, Tae Gyu Lee, In-San Kim, Eun Jung Lee

**Affiliations:** 1 KU-KIST Graduate School of Converging Science and Technology, Korea University, Seoul 02841, Korea; 091989@kist.re.kr; 2 Center for Theragnosis, Biomedical Research Institute, Korea Institute of Science and Technology, Seoul 02456, Korea; t16139@kist.re.kr; 3 Department of Research and Development, ShiftBio, Seoul 02751, Korea; kkksh03@shiftbio.net (S.K.); gbkim@shiftbio.net (G.B.K.); ghnam@shiftbio.net (G.-H.N.); 4 KHU-KIST Department of Converging Science and Technology, Kyunghee University, Seoul 02447, Korea; 5 Department of Chemical Engineering, Kyungpook National University, Daegu 41566, Korea; nykim0310@knu.ac.kr (N.K.); yeram0301@knu.ac.kr (Y.L.); 6 R&D Department Drug Development Division, LabGenomics Corporation, Gyeonggi-do 13488, Korea; lshcommy@gmail.com (S.L.); tekim@labgenomics.com (T.K.); jkhdw@labgenomics.com (K.H.C.); tglee@labgenomics.com (T.G.L.)

**Keywords:** protein nanocage, ferritin, COVID-19, SARS-CoV-2, variants, multivalent vaccine

## Abstract

The SARS-CoV-2 pandemic has created a global public crisis and heavily affected personal lives, healthcare systems, and global economies. Virus variants are continuously emerging, and, thus, the pandemic has been ongoing for over two years. Vaccines were rapidly developed based on the original SARS-CoV-2 (Wuhan-Hu-1) to build immunity against the coronavirus disease. However, they had a very low effect on the virus’ variants due to their low cross-reactivity. In this study, a multivalent SARS-CoV-2 vaccine was developed using ferritin nanocages, which display the spike protein from the Wuhan-Hu-1, B.1.351, or B.1.429 SARS-CoV-2 on their surfaces. We show that the mixture of three SARS-CoV-2 spike-protein-displaying nanocages elicits CD4^+^ and CD8^+^ T cells and B-cell immunity successfully in vivo. Furthermore, they generate a more consistent antibody response against the B.1.351 and B.1.429 variants than a monovalent vaccine. This leads us to believe that the proposed ferritin-nanocage-based multivalent vaccine platform will provide strong protection against emerging SARS-CoV-2 variants of concern (VOCs).

## 1. Introduction

COVID-19 is an infectious disease caused by severe acute respiratory syndrome coronavirus 2 (SARS-CoV-2), which first emerged in Wuhan, China, in 2019. COVID-19 has become a worldwide pandemic, with more than 500 million cumulative cases and nearly 6.2 million deaths as of 14 April 2022 [1]. As a result, worldwide research was mobilized and tremendous efforts were rapidly undertaken to produce SARS-CoV-2 vaccines. This contributed to successful vaccine production, which provided significant protection against the coronavirus. Nevertheless, the virus is constantly changing through various mutations over time. Currently, variants from different countries are prevalently observed, including alpha (B.1.1.7), beta (B.1351), epsilon (B.1429), delta (B.1.617), and omicron (B.1.529). These variants are transmissible [2,3] and immune-resistant, evading antibody responses [4,5], and limit the effects of T-cell immunity [6]. In addition, approved and authorized vaccines have shown a limited ability to prevent infection from these variants, produce lower levels of neutralizing antibodies [7,8], and shorten their effective durability [9,10]. Therefore, the current monovalent vaccines are vulnerable to responses from rapidly changing mutant viruses because of their low cross-neutralization.

Currently, various types of vaccine are available for immunization, including inactivated, vector-based, DNA and mRNA, and peptide and protein subunit vaccines. Conventional recombinant protein-based vaccines have been used for decades and proven to be safe, cost-effective, and simple to manufacture [11]. Moreover, they do not require strict cold-chain supply [12,13]. For SARS-CoV-2 protein-based vaccine candidates, spike proteins and their RBD binding domains are major targets to produce neutralizing antibodies, because they are required for infection by entering, binding, and fusing to angiotensin-converting enzyme 2 (ACE2) on host cells [14]. However, soluble antigens have relatively low stability and tend to induce lower protective immunity, which are the most valuable properties of vaccines [15,16]. Therefore, the design of vaccines with encapsulation and chemical delivery vehicles and the genetic conjugation of antigen to nanocarriers are essential to overcome these limitations. Several approaches, including encapsulation and presenting nanocages, have been utilized to multimerize antigens to improve the production of neutralizing antibodies against subunit vaccine candidates [17,18,19,20].

Ferritin, an intracellular iron-storage protein, self-assembles into a spherical nanocage composed of 24 subunits with high stability. Owing to the capacities that ferritin offers, ferritin nanocages have been proposed in many studies as vaccine platforms against different diseases, including cancer [21,22,23], various viruses [24,25,26], and other pathogens [27]. It has been shown that ferritin nanocages have lymph-node target ability, which makes them able to elicit efficient antigen delivery to antigen-presenting cells (APCs) [22,28], high immunogenicity [27,29], and stability [30,31]. Moreover, the structure of ferritin has the advantage of displaying various antigens by fusing to their surface [27,32]. Therefore, ferritin nanocages could be considered as vaccine candidates for SARS-CoV-2, since they display spike proteins to provoke robust neutralizing antibody responses against multiple virus variant strains.

In this study, we designed a Helicobacter pylori ferritin (HPF)-nanocage-based multivalent vaccine displaying spike proteins from the original SARS-CoV-2 (Wuhan-Hu-1, WT-spike-HPF), B.1.351 (South Africa, V3-spike-HPF), and B.1.429 (California, V4-spike-HPF) variants, which were stabilized to increase immune responses against multiple viruses strains. We evaluated the spike-protein-displaying HPF (spike-HPF) for vaccines against each virus by analyzing the immune profile and cytokine production after vaccination. Moreover, multivalent spike-HPF was compared to monovalent spike-HPF to elicit higher neutralizing antibody titers against variant viruses. This study provides evidence that ferritin-based multivalent vaccines may be potential solutions for preventative medicine against the rapidly changing SARS-CoV-2 virus.

## 2. Results and Discussion

### 2.1. Production and Characterization of SARS-CoV-2 Multivalent Ferritin Nanocage

To develop the ferritin-nanocage-based SARS-CoV-2 vaccine, we chose the HPF nanocage and the spike protein as the nanocarrier and viral target, respectively. Among the various targets of COVID-19 vaccine development, such as spike (S), membrane (M), nucleocapsid (N), and envelope (E) proteins, the spike protein is the main target protein for the COVID-19 vaccine [33]. The spike protein regulates host-cell-receptor recognition and membrane fusion for viral entry with a trimeric structural appearance [34]. In fact, COVID-19 patients have been shown to produce robust neutralizing antibody responses against spike antigens [35]. Importantly, the spike protein of SARS-CoV-2 has shown various mutations throughout its sequence. More specifically, spike protein mutations have been shown to affect viral infectivity and antigenicity [36]. Furthermore, mutations in the spike protein, such as N501Y, E484K, and L452R, may affect viral fitness and transmissibility and, consequently, promote virus infectivity, mortality, and immune escape [37]. Therefore, we developed a multivalent SARS-CoV-2 vaccine against B.1.351 and B.1.429, including N501Y, E484K, and L452R in the spike protein.

**Figure 1:**
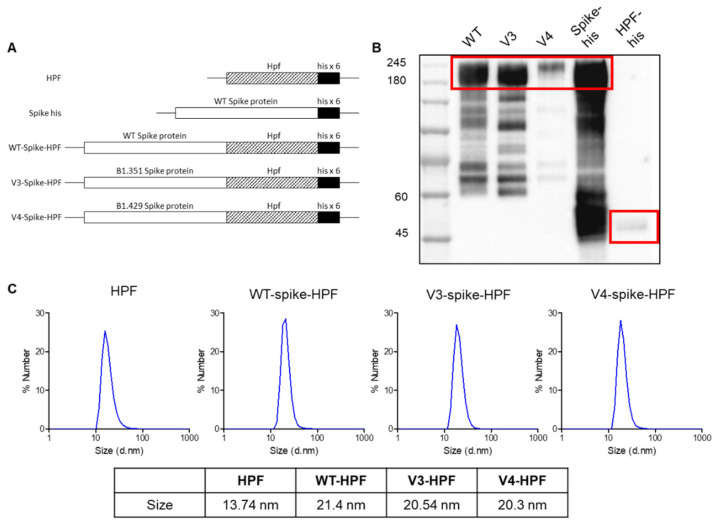
Construct design and characteristics of HPF-based SARS-CoV-2 multivalent vaccines. (**A**) Schematics of HPF, spike, wild type (WT), and variant (V3 and V4) spike-HPF designs. Hexahistidine (his) tag was placed at the C-terminus of the ferritin nanocage. (**B**) The expression of HPF, spike, and spike-HPFs was analyzed by immunoblotting. (**C**) Dynamic light scattering (DLS) patterns of HPF and spike-HPFs with a single peak, and their diameter sizes.

HPF nanocages, which are iron-storage proteins in H. pylori, have been used as effective vaccine delivery vehicles to carry viral immunogens by presenting trimeric glycoproteins on their three-fold axis symmetry [38]. These nanocages showed an especially high level of protective efficiency compared to soluble antigen alone, with minimal risk of autoimmunity through genetic diversity from human ferritin [39]. Additionally, in a very recent phase 1 clinical trial study, no significant effect was found on hematologic parameters, including iron levels, hemoglobin levels, hematocrit, white blood cell counts, and neutrophil counts, after HPF nanocage-derived H2 influenza vaccination [29]. To take advantage of these characteristics, we designed three spike-protein-conjugated HPF nanocages, Wuhan-Hu-1, B.1.351, and B.1.429 plasmids, as immunogens. The spike proteins from Wuhan-Hu-1 SARS-CoV-2 with a histidine tag (Spike-his) and HPF were used as the controls (Figure 1A).

After transiently transfecting the DNA constructs shown in Figure 1A into Expi293F cells, the produced recombinant proteins in the cell culture media were purified through the Ni-NTA protein purification method. The Western blot analysis for the His-tagged spike, WT-HPF, and spike-conjugated HPF confirmed that the recombinant proteins were successfully produced (Figure 1B). The observed molecular weights of the spike-HPFs were larger than the calculated values based on their amino acid sequences due to glycosylation, as described in previous reports [24,40]. Although the spike-HPFs showed an array of bands below the main band of the recombinant protein expressed due to degradation in the reducing conditions, the sizes of the majority of the bands were as expected (~200 kDa). The purified HPF nanocages decorated with the spike proteins were monodispersed in size, according to the DLS analysis. In addition, the spike-conjugated HPF showed a slightly larger size (~21 nm), compared to the WT-HPF (~13 nm) (Figure 1C). These results showed that the desired spike antigens were expressed on the surfaces of the self-assembled ferritin nanocages.

### 2.2. Activation of Immune Response of SARS-CoV-2 Multivalent Ferritin Nanocage

**Figure 2:**
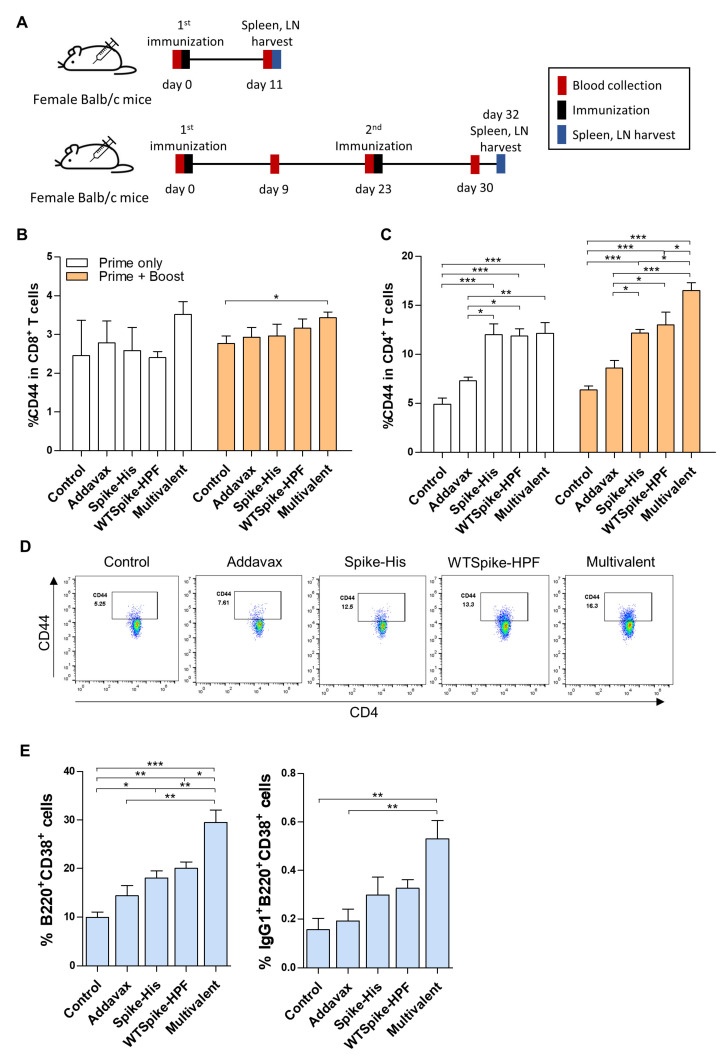
Evaluation of immune responses post-multivalent spike-HPF vaccine immunization. (**A**) Immunization schedule, including primary and booster injection of spike-HPF. Animals were immunized with 50 μg of antigens at day 0 and 23. At day 11 and 30, spleen and lymph nodes were harvested, and the immune profiles were analyzed. (**B**–**D**) Antigen-experienced memory T cells in lymph nodes were increased after spike-HPF immunization. The expressions of CD44 in (**B**) CD8^+^ T cells and (**C**,**D**) CD4^+^ T cells were analyzed by flow cytometry. (**E**) Protection mediating B cells were expanded in lymph nodes upon multivalent spike-HPF vaccination. Activation (left) and memory (right) markers expressing B cells were analyzed using flow cytometry. Statistical comparisons were performed using Student t-test and statistically significant differences were presented as follows: *p* < 0.05 *, *p* < 0.01 **, or *p* < 0.001 ***.

To assess the efficacy of the ferritin-nanocage-based multivalent vaccines, we investigate the activation of CD4^+^-T-cell, CD8^+^-T-cell, and B-cell immune responses in vivo. Monovalent vaccine (WT-spike-HPF), multivalent vaccine (WT-spike-HPF + V3-spike-HPF + V4-spike-HPF), soluble spike-his, adjuvant (Addavax), or PBS were injected into mice, following the schedule in Figure 2A. The mono- or multivalent vaccines were injected subcutaneously in a 1:1 mixture with the adjuvant, AddaVax^TM^.

As shown in Figure 2B,C, the primary–booster immunization strategy with the multivalent ferritin nanocage showed a significantly effective CD4^+^-T-cell and CD8^+^-T-cell immune response in the lymph nodes (LNs). We observed an increasing trend in CD44 expressions as a marker of antigen experience in the CD8^+^ T cells, which mounted a protective response against the SARS-CoV-2 (Figure 2B, Supplementary Figure S1A) [41]. Importantly, the multivalent vaccine treatment significantly upregulated the percentage of CD44 in the CD4^+^ T cells, as well as the quantification of the CD44^+^CD4^+^ T cells in the LNs (Figure 2C,D, Supplementary Figure S1B). It has been reported that CD4^+^-T-cell immunity appears to be essential for the proper control of COVID-19 [42]. In addition, CD4^+^ T cells play a significant role in orchestrating cellular and humoral immunity [43]. In a previous study, a ferritin-based SARS-CoV-2 monovalent vaccine promoted CD4^+^-T-cell immunity more strongly than CD8^+^-T-cell immunity [44]; similarly, the ferritin-based multivalent vaccine proposed in this study also induced CD4^+^-T-cell immunity more strongly than CD8^+^-T-cell immunity.

In addition, the number of B220^+^CD38^+^ B cells, which mainly act as memory B cells, was highly increased in the LNs of the mice in the multivalent-vaccine-treated group. In a similar context, IgG1^+^B220^+^CD38^+^ cells capable of inducing antibody formation were significantly expanded in the multivalent-vaccine-treated group compared to the other control groups (Figure 2E). Collectively, these findings suggest that the engineered ferritin-nanocage-based multivalent vaccine efficiently upregulates T- and B-cell immune responses.

### 2.3. SARS-CoV-2 Variant-Specific Immune Response of Multivalent Ferritin Nanocage

**Figure 3:**
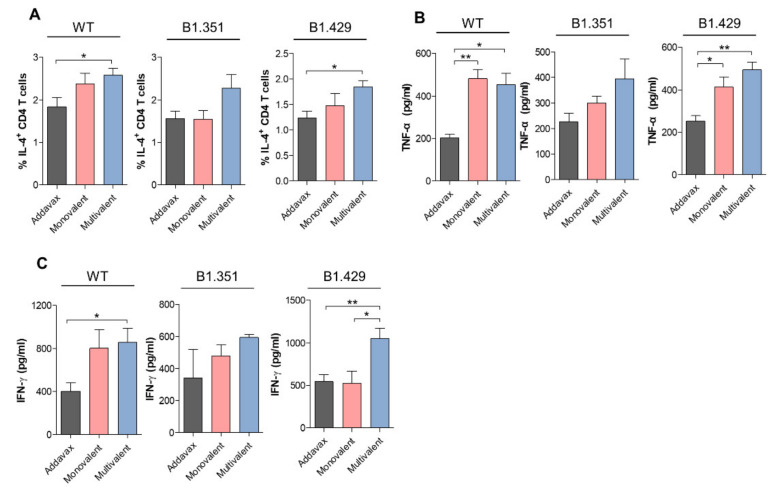
SARS-CoV-2 WT and variant-specific immune responses induced by multivalent spike-HPF vaccine. Immune cells were stimulated with peptide pools corresponding to the fragments of WT, B1.315, and B1.429. (**A**) The proportion of IL-4-producing SARS-CoV-2 WT and variant-specific CD4^+^ T cells, indicating that Th2 responses were detected using flow cytometry. (**B**,**C**) Cytokine secretion from peptide-stimulated CD4^+^ T cells was determined by ELISA. (**B**) TNF-α and (**C**) IFNγ, indicating that Th1 responses were evaluated. Statistical comparisons were performed using a Student t-test, and statistically significant differences were presented as follows: *p* < 0.05 * or *p* < 0.01 **.

To confirm the variant-specific immune response following the multivalent ferritin nanocage vaccination, we analyzed the cytokine profiles from the CD4^+^ T cells under B1.351 and B1.429 spike antigen challenges [45]. First, the IL-4 expressing CD4^+^ T cells in the spleen were analyzed to evaluate the Th2 response directly affecting the B-cell immune response by forming neutralizing antibodies. Briefly, single cell-digested spleen cells were cultured with SARS-CoV-2 spike peptide pools of the wild type (WT) and each variant. As shown in Figure 3A, a proportion of the IL-4^+^CD4^+^ T cells significantly increased in the multivalent-ferritin-nanocage-vaccine-treated group when compared to the adjuvant-treated control under the wild-type and B1.429 spike-peptide treatments. BY contrast, the monovalent-vaccine-treated group showed no significant change. In addition, the stimulation with the SARS-CoV-2 spike peptide pool of the B1.351 variant showed an increasing trend in IL-4^+^CD4^+^-T-cell proportion in the spleens of the mice treated with the multivalent vaccine, whereas the monovalent-vaccine-treated group did not show any difference in IL-4^+^CD4^+^-T-cell proportion.

To address the Th1 immune response against the vaccinations, the cytokines secreted from the CD4^+^ T cells from the digested spleen, such as TNFα and IFNγ [46], were also analyzed with an enzyme-linked immunosorbent assay (ELISA). Under the challenge of the WT spike peptide pool, both the monovalent (wild type) and the multivalent (wild type, B1.351 and B1.429)-vaccine-treated groups showed a similar increase in the amount of TNFα and IFNγ to the adjuvant-treated control (Figure 3B,C). The monovalent-vaccine-treated group also showed an increasing trend when challenged with the B1.351 or B1.429 spike peptide pool, possibly due to cross-reactivity. However, although the increase in the case of B.1.351 was not statistically significant, the multivalent-vaccine-treated group showed a tendency to increase TNFα secretion and a stronger IFNγ secretion from the CD4^+^ T cells than the monovalent-vaccine-treated group. In particular, it is noteworthy that IFNγ secretion is a key cytokine in several antiviral responses and has been reported to inhibit SARS-CoV replication by synergizing with type I interferons [47,48]. In the case of B1.429, the IFNγ secretion was increased only in the multivalent-vaccine-treated group, thereby showing a lack of cross-reactive immune response formation between the WT and the B1.429 variant in the monovalent vaccine treatment. Overall, the results suggest the potency of ferritin nanocage-based multivalent vaccine in inducing B1.351 and B1.429 antigen-specific Th1 and Th2 immune responses.

### 2.4. SARS-CoV-2 Variant-Specific Antibody Formation of Multivalent Ferritin Nanocage

**Figure 4:**
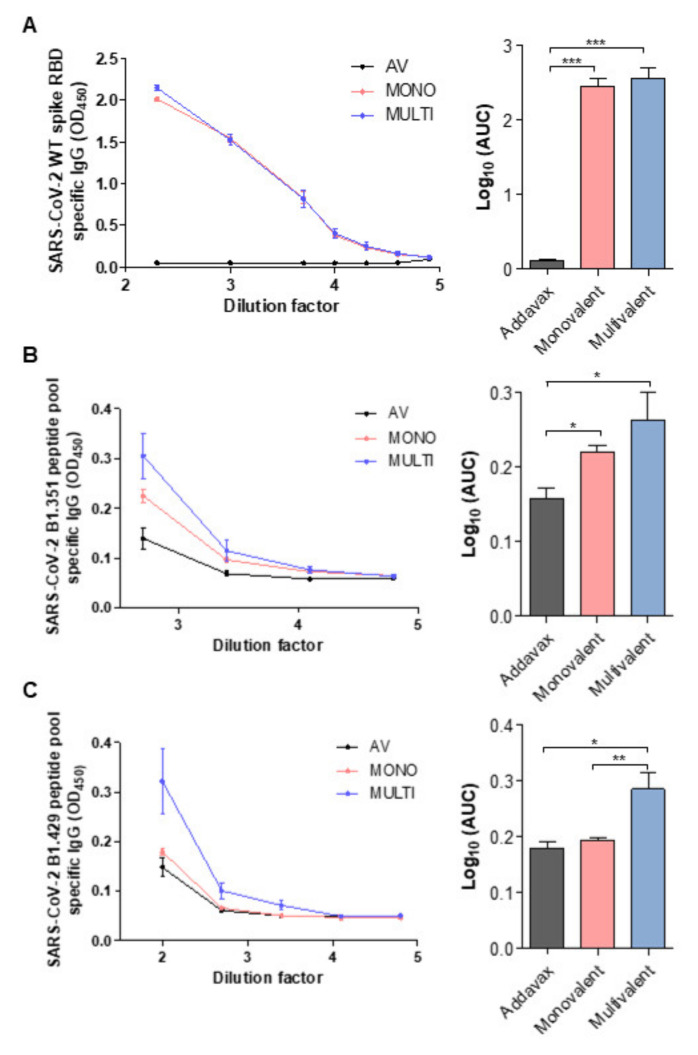
WT-, B1.315-, and B1.429-specific IgG responses following immunization with mono- and multivalent vaccine. (**A**–**C**) ELISA was performed using serially diluted serum samples from immunized animals. (**A**) WT-, (**B**) B1.315-, and (**C**) B1.429-specific antibody titers were quantified by area under the curve (AUC). Statistical comparisons were performed using Student t-test and statistically significant differences were presented as follows: *p* < 0.05 *, *p* < 0.01 **, or *p* < 0.001 ***.

Next, we evaluated the efficacy of the multivalent ferritin nanocage vaccine strategy by measuring IgG specificity to the spike protein of SARS-CoV-2. The titers of antibodies in the collected blood serum from each group were analyzed by ELISA. Briefly, the SARS-CoV-2 WT and variants’ antigen-specific antibodies were captured with the receptor-binding domain (RBD) of the WT spike protein or with the immunogenic peptide pools of B1.351 and B1.429, respectively. When captured with the WT RBD domain, the serum dilution factor and AUC showed that both the monovalent and the multivalent vaccination generated considerable WT-specific antibodies compared to the adjuvant-treated control (Figure 4A).

Furthermore, with the B1.351 spike peptide pool, the monovalent- and multivalent-vaccine-treated groups showed higher antigen-specific antibody concentrations than the adjuvant-treated group. This demonstrates the ability of the monovalent vaccine to produce cross-reactive antibodies against the B1.351 peptide pool (Figure 4B). Although not statically significant, the multivalent-vaccine-treated group showed an increasing trend of antigen-specific antibody formation compared with the monovalent-vaccine-treated group. Importantly, the B1.429 peptide pool-specific antibodies were generated only with multivalent vaccination, not with monovalent vaccination (Figure 4C), which was consistent with the results of the IFNγ secretion in the CD4^+^ T cells (Figure 3C). Altogether, these results suggest that ferritin-nanocage-based multivalent vaccines may protect against SARS-CoV-2 variants.

## 3. Materials and Methods

### 3.1. Animals

Seven-to-eight-week-old female Balb/c mice were obtained from Orient Bio. (Gyeonggi-do, Korea) and housed in an animal facility of the Korea Institute of Science and Technology (KIST). On day 0, the animals were randomly dispatched into four groups, and vaccines were subcutaneously injected for prime injection; they were subsequently injected with a booster shot on day 23. The animals were sacrificed on day 32 after the final vaccination. The blood was collected, and the spleens and lymph nodes were harvested for immune profile analysis. All the animal experiments were performed with the approval of the Institutional Animal Care and Use Committee (IACUC) of KIST.

### 3.2. DNA Construct (Cloning)

Spike protein variant genes were purchased from InvivoGen. All primers used in this study are listed in Table 1. PCR was performed using Phusion™ Hot Start II DNA Polymerase (Theromofisher Science, Waltham, MA, USA, F549XL), following the manufacturer’s protocol. In-Fusion HD cloning kit (Clontech, Mountain View, CA, USA, 639649) was used to clone the various spike-ferritin vectors. Ferritin vector was linearized by PCR and purified by a QIAquick PCR purification kit (Qiagen, Limburg, The Netherlands, 28106). Spike protein PCR primers for In-Fusion cloning contained sequences that were complementary to the ends of the linearized vector. Spike protein PCR products proceeded in the same way as ferritin vector. The mixture was made by the molar ratio of spike protein PCR product and ferritin vector, as recommended by protocol. Spike protein vector was cloned by restriction digest and ligation. The spike protein PCR primers contained BamHI-HF (NEB, Ipswich, MA, USA, R3136S) or XhoI (NEB, R0146S) site. The digestion of each vector and spike protein PCR product was incubated at 37 °C for 1 h. Quick CIP (NEB, M0525S) was added to the vector reaction only for 10 min. Next, agarose gel purification to remove enzymes and fragments, followed by a QIAquick gel extraction kit (Qiagen, 28704), was performed. After gel purification, the vector and PCR product were mixed and ligated with T4 DNA ligase (Roche, Basel-Stadt, Switzerland, 10716359001) at RT for 1 h. The mixture was used for the transformation of Escherichia coli TOP10 competent cells. All final cloning constructs were confirmed by DNA sequencing (CosmoGentech Co., Seoul, Korea).

### 3.3. Cell Culture, Protein Expression, and Purification

Expi293F cells (ThermoFisher) were cultured in Expi293 expression medium (ThermoFisher) at 37 °C with 8% CO_2_ on an orbital shaker. Cells were sub-cultured when the density reached 1–3 × 10^6^ viable cells/mL. For protein expression, all plasmid vectors were transiently transfected into Expi293F cells using ExpiFectamin 293 reagent (ThermoFisher) with Opti-mem (Gibco, Waltham, MA, USA) complexation buffer. One day post-transfection, transfection enhancers were added to the transfection flasks. Three-to-six days post-DNA transfection, the supernatant was collected via centrifugation at 3500× *g* for 30 min and debris were removed through 0.22-micrometer filtration. Using Ni-NTA chromatography and agarose bead (Qiagen), His-tagged HPFs were purified.

### 3.4. Characterization

Purified spike conjugated ferritin nanocage samples were quantified by bovine serum albumin assay. For Western blot, equal amounts of each sample were loaded into SDS PAGE. The SDS-PAGE was then transferred into a nitrocellulose membrane, which was blocked for 1 h in 0.5% skim milk/TBS-T. Subsequently, the membrane was incubated with a primary antibody specific for His-tag at 4 °C overnight. The next day, after washing with TBS-T (0.05% Tween-20), the membrane was stained with HRP-conjugated secondary antibodies for 1 h at room temperature. Finally, the results were visualized with ChemiDoc MP Imaging System (BioRad, Hercules, CA, USA). The size of purified spike conjugated ferritin nanocages was analyzed by Zetasizer Nano ZS (Malvern Instrument, Malvern, UK).

### 3.5. Immune Profile Analysis

Spleen and draining lymph nodes were harvested 2 weeks after the final vaccination. Harvested spleens were digested with gentle MACSTM Octo Dissociator (Miltenyi Biotec, Bergisch Gladbach, Germany) and lymph nodes were manually ground to single-cell suspension, which was then RBC-lysed with RBC lysis buffer (Biolegend, San Diego, CA, USA) for 5 min at 4 °C. Single-cell suspension of lymph nodes was stained with immune-cell-specific antibodies for flow cytometry (BD Accuri C6, BD Bioscience, Franklin Lakes, NJ, USA) as follows: PERCP anti-CD45.2 antibody, APC anti-CD3 antibody, FITC anti-CD4 antibody for CD4 T cell; PERCP anti-CD45.2 antibody, APC anti-CD3 antibody, FITC anti-CD8 antibody for CD8 T cell; and PERCP anti-CD45.2 antibody, APC anti- antibody, FITC anti- antibody, PE anti-B220 antibody for B cell.

Single-cell suspension of spleen was seeded into a 96-well plate and challenged with SARS-CoV-2-spike-protein–peptide-pool (Miltenyi peptide pool; 130-127-958, 130-128-482). After incubating for 5 h, the supernatant was collected for TNFα and IFNγ ELISA (R&D systems, Minneapolis, MN, USA), which were performed according to the manufacturer’s protocol. The cells were centrifuged to stain with PE-conjugated anti-IL-4 antibody (Biolegend).

### 3.6. Neutralizing Antibody ELISA

The blood from the vaccinated mice was collected through the retro-orbital sinus. Serum was isolated by immediate centrifugation (13,000 rpm, 4 °C) after collecting the blood. Next, the serum was diluted to identify the titer of the neutralizing antibody with antigen-specific ELISA kit. Wild-type spike RBD domain-specific ELISA kit (RAS-N031) was purchased from Acrobiosystems (Newark, NJ, USA).

For B1.351- and B1.429-variant spike-protein-specific ELISA, SARS-CoV-2-spike-protein–peptide-pool (Miltenyi peptide pool; 130-127-958, 130-128-482) was coated onto Nunc Maxisorp^TM^ flat-bottom (ThermoFisher, Waltham, MA, USA). Next, the diluted serum was added, which was captured by Peroxidase AffiniPure Goat anti-mouse IgG (H + L) (Jackson Immuno Research, Chester County, PA, USA).

### 3.7. Statistics

All the experiments were repeated at least three times with two to four biological replicates. All the data produced were statistically analyzed through Prism 5 software. Data are presented with means ± standard error of the mean (SEM), denoted by error bars. Statistical significance was calculated by one-way ANOVA with Tukey’s post hoc test or Student’s t-test. The significance is indicated as follows: *p* < 0.05 *, *p* < 0.01 **, or *p* < 0.001 ***.

## 4. Conclusions

Protein nanocages have been used intensively in the biomedical field and in nanotechnology, including in imaging, drug delivery, and vaccine development, because of their characteristics [28,49,50,51]. They are highly biocompatible and homogenous assemblies, which are suitable for bioengineering via both genetic and chemical modification [49]. The main advantage of protein nanocages as vaccine platforms is their ability to display a variety of desired antigens on their surfaces [52]. In addition, it has been indicated that the multivalent presentation of antigens could elicit robust immune responses, including the retention of follicular dendritic and helper T cells and the formation and expansion of B cells [53]. Therefore, several protein-nanocage-based vaccines expressing antigens of interest have been actively explored as efficient vaccine platforms in clinical vaccine development [54].

In particular, ferritin is a naturally existing iron-storage nanocage that self-assembles with spherical particles and is highly stable under various physiological conditions [55]. Therefore, ferritin nanocages have been used as vaccine vehicles for various diseases and are currently being evaluated as vaccine platforms against Influenza and Epstein–Barr virus (NCT03186781, NCT03814720, and NCT04645147). In particular, ferritin-nanocage-based COVID-19 vaccines are attracting attention as powerful platforms because of their structural stability and potent antiviral immune responses [24,56]. Furthermore, it is known that ferritin-nanocage-based SARS-CoV-2 spike vaccines show broad neutralizing antibody responses and have a higher maximum titer than others [57,58]. The multiple intrinsic advantages of ferritin nanocages provide a strong motive to develop them into potent COVID-19 vaccine platforms. The SARS-CoV-2 vaccine based on the ferritin nanocage expressing Wuhan Hu-1 spike protein (NCT04784767) is currently entering phase 1 clinical trials.

In this study, we simultaneously developed a ferritin-nanocage-based vaccine targeting three different SARS-CoV-2 variants. It efficiently enhanced the T- and B-cell immune responses, especially the CD4^+^-T-cell immune response, rather than the CD8^+^-T-cell immune response, similar to the result of the ferritin-nanocage-based monovalent vaccine. Although monovalent vaccines also showed an increasing trend toward Th1 and Th2 immune response against the B1.351 and B1.429 spike antigens by cross-reactivity, the proposed multivalent vaccines showed much more robust responses. Similarly, in SARS-CoV-2-variant-specific antibody formation, it was confirmed that the proposed multivalent vaccine showed a much more potent antibody formation induction ability than the monovalent vaccines. Importantly, for the B.1.429 variant, the monovalent vaccines did not show antigen-specific IFNγ formation and failed to form antibodies f, but the proposed multivalent vaccine showed potent antigen-specific CD4^+^-T-cell and antibody formation against B.1.429.

Thus, utilizing the easily manufacturable protein-nanocage-based vaccine platform, we demonstrated that the proposed SARS-CoV-2 multivalent vaccine, a mixture of three different variant-targeting HPF-conjugates, could elicit variant-specific immune response and antibody formation. Although only the B.1.351 and B.1.429 variants were used in this study, they may be readily applicable to other SARS-CoV-2 VOCs, including omicron.

## Figures and Tables

**Table 1:** List of primers used in this study.

Template	Primer Sequence
Ferritin vector	Forward 5′-AGCTCGGATCCGATGTTATCAAAAGACA Reverse 5′-CAAGCTTCGTACGGCGCGC
Spike WT vector Spike V3 vector	Forward 5′-GCCGTACGAAGCTTGCTGGTCAGTTCCCA Reverse 5′-CATCGGATCCGAGCTTCCAAGCTCCTCCTTGAA
Spike V4 vector	Forward 5′-GCCGTACGAAGCTTGCTGGTCAGTATCCAATG Reverse 5′-CATCGGATCCGAGCTTCCAAGCTCCTCCTTGAA
Spike WT vector	Forward 5′-AAAAAGGATCCACTGGTCAGTTCCCAA Reverse 5′-TTTTTCTCGAGAAAGCTCCTCCTTGAA

## Data Availability

All the data presented in this study are available in this article.

## Supplementary Materials

The following supporting information can be downloaded at: https://www.mdpi.com/article/10.3390/ijms23116123/s1.
