# Supplementary material for: A Multivalent Vaccine Based on Ferritin Nanocage Elicits Potent Protective Immune Responses against SARS-CoV-2 Mutations
Source: Int J Mol Sci. 2022 May 30;23(11):6123. doi: 10.3390/ijms23116123 (PMC9181758; doi:10.3390/ijms23116123)
Supplement: Supplementary file 1 [file ijms-23-06123-s001.zip › ijms-1740568-supplementary.pdf]

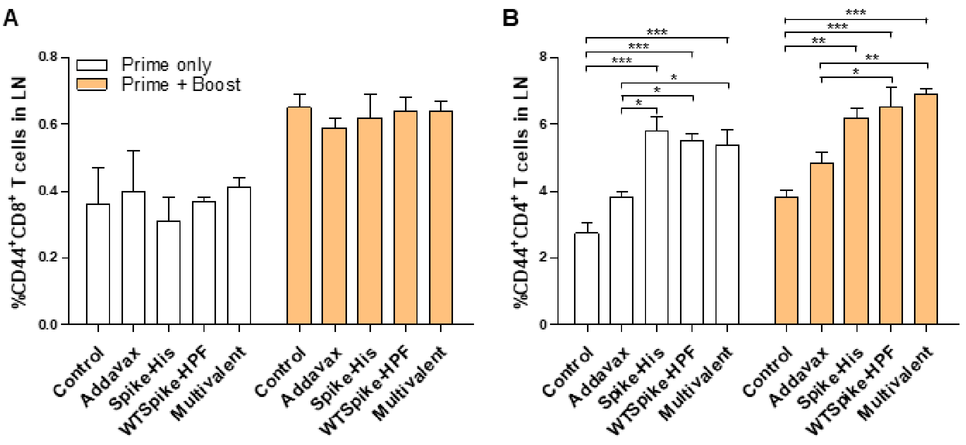

Figure S1. The memory T cells in lymph nodes upon HPF-Spike vaccination. (**A**,**B**) CD44 expressing $CD8^+$ (**A**) and $CD4^+$ (**B**) T cells were analyzed using flow cytometry. Statistical comparisons were performed using student t-test and statistically significant differences were presented as follows: $p < 0.05$ *, $p < 0.01$ **, or $p < 0.001$ ***.
